# A Novel Gene CDC27 Causes SLE and Is Associated With the Disease Activity

**DOI:** 10.3389/fimmu.2022.876963

**Published:** 2022-03-28

**Authors:** Shunlai Shang, Yena Zhou, Keng Chen, Lang Chen, Ping Li, Diangeng Li, Shaoyuan Cui, Mei-Jun Zhang, Xiangmei Chen, Qinggang Li

**Affiliations:** ^1^ School of Medicine, Nankai University, Tianjin, China; ^2^ Department of Nephrology, The First Medical Center, Chinese PLA General Hospital, Medical School of Chinese PLA, Chinese PLA Institute of Nephrology, State Key Laboratory of Kidney Diseases, National Clinical Research Center for Kidney Diseases, Beijing, China; ^3^ Clinical Medical School, Guangdong Pharmaceutical University, Guangzhou, China; ^4^ Medical Technology & Bioinformatics Department, Beijing Mygenostics co., LTD, Beijing, China; ^5^ Department of Academic Research, Beijing-Chaoyang Hospital, Capital Medical University, Beijing, China; ^6^ Bioinformation Department, Geneis (Beijing) Co., Ltd., Beijing, China

**Keywords:** lupus family, multiple bioinformatics analysis, CDC27, whole exone sequencing, marker

## Abstract

**Background:**

As genetic genetic factors are important in SLE, so screening causative genes is of great significance for the prediction and early prevention in people who may develop SLE. At present, it is very difficult to screen causative genes through pedigrees. The analytical method described herein can be used to screen causative genes for SLE and other complex diseases through pedigrees.

**Methods:**

For the first time, 24 lupus pedigrees were analyzed by combining whole exon sequencing and a variety of biological information tools including common-specific analysis, pVAAST (pedigree variant annotation, analysis and search tool), Exomiser (Combining phenotype and PPI associated analysis), and FARVAT (family based gene burden), and the causative genes of these families with lupus identified. Selected causative genes in peripheral-blood mononuclear cells (PBMCs) were evaluated by quantitative polymerase chain reaction (qPCR).

**Results:**

Cell division cycle 27 (CDC27) was screened out by common-specific analysis and Exomiser causative gene screening. FARVAT analysis on these families detected only CDC27 at the extremely significant level (false discovery rate <0.05) by three family-based burden analyses (BURDEN, CALPHA, and SKATO). QPCR was performed to detect for CDC27 in the PBMCs of the SLE family patients, sporadic lupus patients, and healthy people. Compared with the healthy control group, CDC27 expression was low in lupus patients (familial and sporadic patients) (*P*<0.05) and correlated with lupus activity indicators: negatively with C-reactive protein (CRP) (*P*<0.05) and erythrocyte sedimentation rate (*P*<0.05) and positively with complement C3 and C4 (*P*<0.05). The CDC27 expression was upregulated in PBMCs from SLE patients with reduced lupus activity after immunotherapy (*P*<0.05). Based on Receiver operating characteristic (ROC) curve analysis, the sensitivity and specificity of CDC27 in diagnosing SLE were 82.30% and 94.40%.

**Conclusion:**

The CDC27 gene, as found through WES combined with multiple analytical method may be a causative gene of lupus. CDC27 may serve as a marker for the diagnosis of SLE and is closely related to the lupus activity. We hope that the analytical method in this study will be used to screen causative genes for other diseases through small pedigrees, especially among non-close relatives.

## 1 Background

Systemic lupus erythematosus (SLE) is a chronic systemic inflammatory disease that can affect many organs, such as the kidneys, lungs, and skin (1, 2). Prediction and early prevention are of great significance for people who are likely to develop SLE (1). Although the etiology of SLE remains unclear, genetic factors are thought to be important for its occurrence (3). More than 5% of cases of lupus are familial, and the agreement rate between identical twins is 40% (4). Moreover, onset of lupus shows a familial aggregation, and the heritability rate is estimated to be 44-66% (5–7). The risk of SLE increases significantly in individuals with first-degree relatives with SLE (3). SLE has obvious genetic clustering and genetic predisposition, which strongly suggests the important role of genetic factors in its pathogenesis (7, 8).

Compared with traditional research methods for single-gene diseases, whole-exome sequencing (WES) has the advantages of being less time-consuming and having high throughput, low sample requirements, high sensitivity, and low cost (9). WES improves the efficiency of causative gene discovery at lowcost (10), which is important for the diagnosis, treatment, and prevention of many diseases (11–15).

In this study many lupus families (24 families) were for the first time evaluated by combining WES with multiple analytical methods, and possible causative genes of SLE were identified,enriching our knowledge on the causative mechanism of SLE. This study identified cell division cycle 27 (CDC27) as a possible causative gene for SLE. Quantitative polymerase chain reaction (qPCR) showed that compared with healthy individuals, the expression trend of CDC27 in patients with sporadic lupus was the same as that in familial patients, indicating that CDC27 might serve as a marker for the diagnosis of SLE. CDC27 is a component of the anaphase-promoting complex (APC), is an E3 ubiquitin ligase that catalyzes ubiquitin-mediated proteasomal degradation of type B cell cycle proteins and stimulates the cell cycle transition from the middle stage to the later stage (16, 17). Abnormal CDC27 expression is associated with autoimmune diseases (18) and cancer (19), and it can help significantly in predicting breast cancer recurrence (20). No study to date has reported familial aggregation of CDC27 in specific diseases, nor has the direct relationship between abnormal CDC27 expression and the pathogenesis of SLE been investigated. We hope that this study will improve the utility of CDC27 in the diagnosis of SLE.

## 2 Experimental Materials and Methods

### 2.1 Family Ascertainment

The subjects involved in this study met the research classification criteria of the 2012 Systemic Lupus Collaborating Clinics for SLE. On the one hand, familial lupus patients were included. Familial SLE was defined as SLE confirmed in at least two relatives. The family members of the lupus patients were all nonconsanguineous members, and the healthy members of the family were studied as the familial control group. Patients with sporadic lupus were also included. The above patients were all admitted to the People’s Liberation Army General Hospital between December 2016 and December 2020. The healthy control group for sporadic patients were volunteers from the same hospital with normal physical examination at the same time. Voluntary, written, fully informed consent was obtained from all participants.

### 2.2 DNA Extraction, Library Preparation, and Whole-Exome Sequencing

#### 2.2.1 DNA Extraction

Peripheral-blood samples were collected from participating subjects, and genomic DNA was obtained using the TIANamp genomic DNA kit (Catalog no. DP304; TIANGEN) according to the manufacturer's protocol.

#### 2.2.2 Exon Library Preparation

The genomic DNA template was used for library preparation (small fragments) according to the method and procedure of SureSelectXT Target Enrichment System (G7530-90000), and followed by hybridization and capture.

#### 2.2.3 Sequencing

For qualified libraries, paired-end sequencing (PE150) was performed on the HiSeq sequencing platform to obtain 150-bp sequence reads. Exon data of each sample (25-30 Gbp) were sequenced at a depth of 200X.

### 2.3 Bioinformatic Analysis

**Figure 1:**
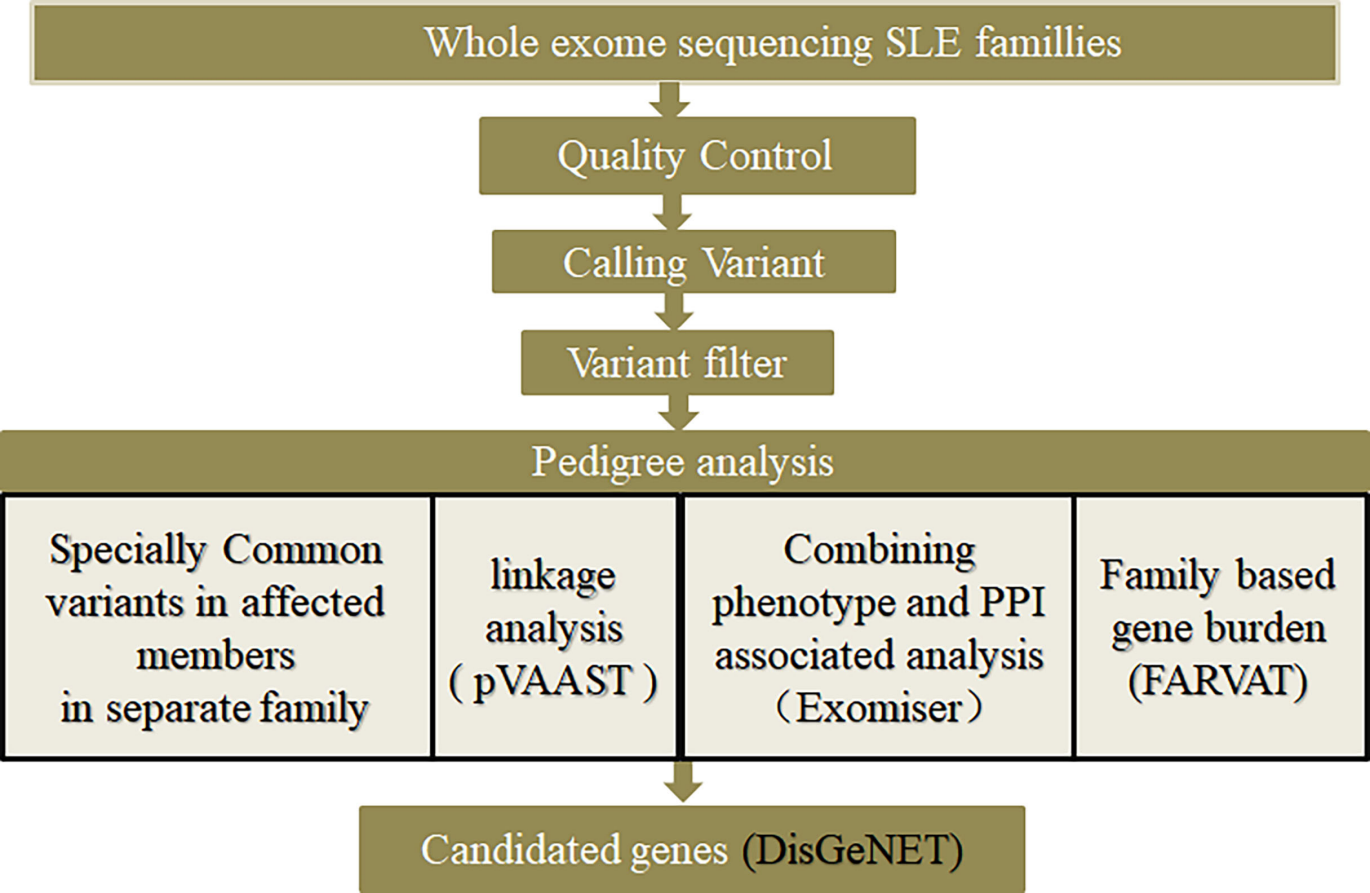
Diagram of bioinformatic analysis.

The families with lupus were divided according to whether male family members had the disease, into class 1 (families with both male and female members having the disease) and class 2 (families with female members having the disease, but without it being clear whether male members would develop have the disease in the future). The bioinformatic analysis scheme of this study is shown in **Figure 1**.

#### 2.3.1 Data Quality Control

Sequencing data were quality-controlled with adapter and aligned to human reference genome build hg19 (ftp://hgdownload.soe.ucsc.edu/goldenPath/hg19/) with Burrows–Wheeler Aligner (bwa) software version 0.7.12-r1039. The aligned reads were sorted and indexed using SAMtools version 1.2.1, and the Genome Analysis Toolkit (GATK, version 3.8) was used to realign reads to the genome and eliminate PCR duplicates. The GATK4.0 best practice haplotype caller pipeline was used for SNP and indel calling. All SNPs were annotated using ANNOVAR and related database download at Feb. 20.2020 Several genomic databases, including 1,000 Genomes (1000G), ExAC (Exome Aggregation Consortium), Exome Sequencing Project (ESP), gnomAD (both WES and WGS databases) (v2.1.1), and CG46, were used to assess the variant frequency in the population. MCAP(v1.3), SIFT, Polyphen2-HDIV, Polyphen2-HVAR, MutationTaster, MutationAssessor, and Clinvar (version 20200316) were used to annotate the effect of missense variants. GERPs were used to evaluate the conservation of the variant locus.

#### 2.3.2 Data Analysis

Considering the importance of sexfor onset (21–27), lupus families were divided into two types: class 1 and class 2. Preliminary screening of causative genes and related loci was performed using three methods based on variant loci (common-specific analysis; Pedigree Variant Annotation, Analysis, and Search Tool (pVAAST) multiline linkage and correlation analysis; Exomiser causative gene screening). As a method in pedigree studies, gene-based FARVAT was also used to perform gene burden analysis, which directly considers some genes with weak single-locus effect values and low single-locus mutation frequencies, in order to supplement the above three methods. The candidate genes screened by the above methods were annotated in the DisGenet database.

##### 2.3.2.1 Pedigree Screening for Common-Specific Analysis in Affected Members

**Figure 2:**
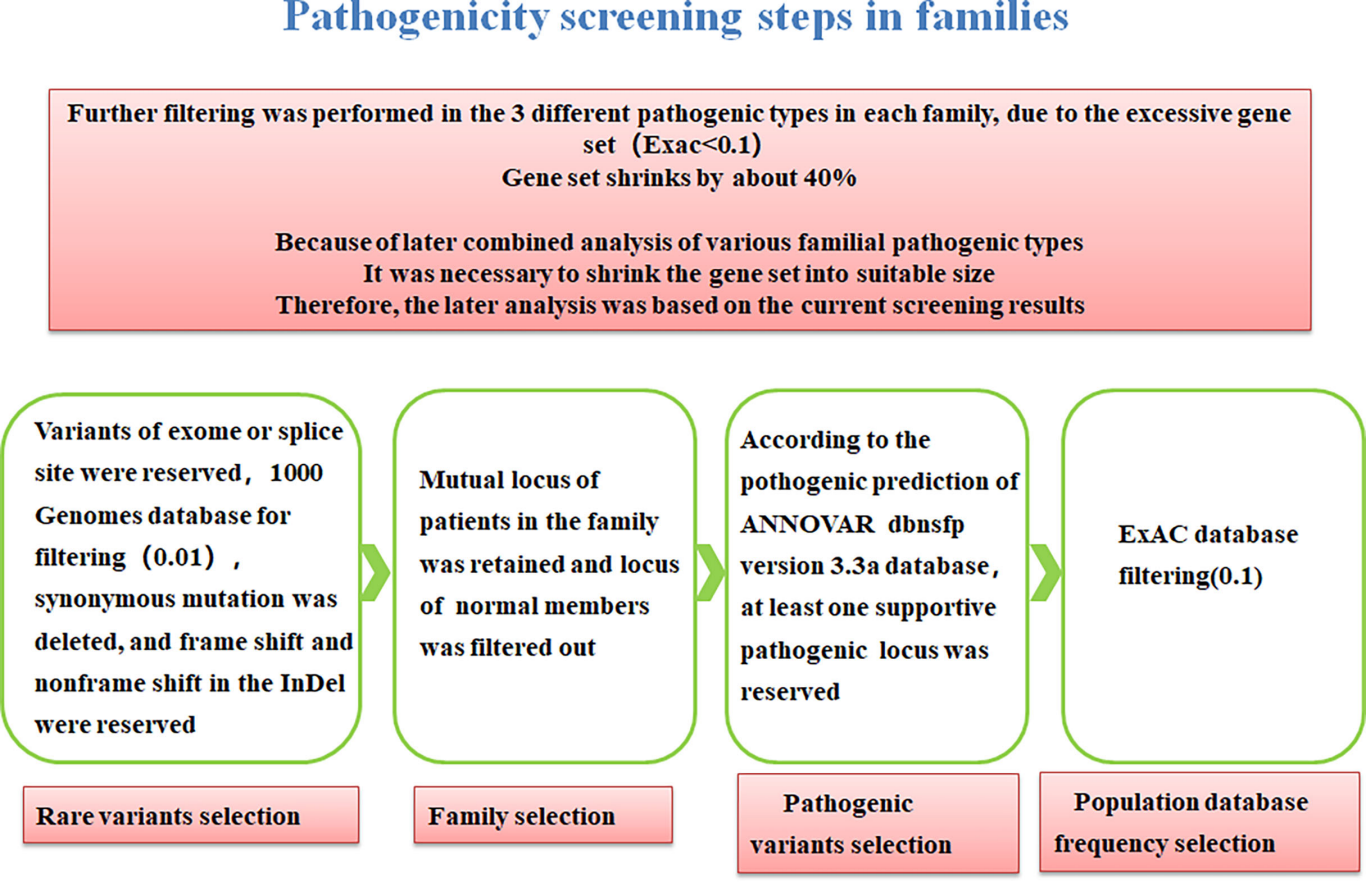
Procedure of common-specific analysis.

We screened the families with multiple affected individuals as well as healthy family members and there is procedure of common-specific analysis in **Figure 2** (28).

##### 2.3.2.2 pVAAST Multiline Linkage and Correlation Analysis

pVAAST combines two different statistical methods used for identifying disease-causing gene mutations (29). This combination approach outperforms individual familial analytical methods by increasing the power or speed with which mutations are identified and reducing complications through study design and analysis. pVAAST is robust with regard to incomplete penetrance and locus heterogeneity and is applicable to a variety of genetic traits. pVAAST performs well in research on a single gene or a highly permeable phenotype in a single lineage or in research on a highly polygenic and common phenotype involving hundreds of lineages.

##### 2.3.2.3 Human Phenotype Ontology tTerm Conversion and Exomiser CausativeGene Screening in Diseased Samples

Exomiser uses random-walk analysis of protein interaction networks, clinical correlation and cross-species phenotype comparison, and various other computational filters (for variable frequency, pathogenicity prediction, and lineage analysis) to prioritize exon sequences (30). Exomiser can detect potential disease-causing variants in one exon or a whole genome. It mines causative genes and variant loci based on mutation frequency, pathogenicity, mutation quality, genetic pattern, and HPO phenotype data. Based on the Exomiser screening results, the genes with an Exomiser score, Phenotype score, and Variant score all of 0 were filtered out and combined with the annotation results of the variant loci by Exomiser. Nonpathogenic loci were filtered out (definition of causative locus: any one of CADD, POLYPHEN, MUTATIONTASTER, and SIFT identified it as pathogenic, or it had not been annotated).

##### 2.3.2.4 FARVAT: Family Based Correlation Analysis

FARVAT, a family-based rare variant association test, can be used to study extended families (31). The software combines the burden test and the variant component test and employs three types of parameters to perform the correlation test. When all the loci in a certain region are pathogenic, the BURDEN parameters perform the best; when some of the loci in a certain region are causative, the CALPHA parameters perform the best; SKATO combines the statistical strategies of the former two to achieve very robust performance. FARVAT uses only SNPs for analysis. If a sample contains only one parent and the other parent is missing, then this sample is excluded from analysis. Note: The software can obtain the *p* values of three parameters, and any one parameter with *P<*0.05 is a candidate.

##### 2.3.2.5 Annotation of Candidate Genes in the DisGenet Database

DisGeNET (Janet Piñero, Núria Queralt-Rosinach, Àlex Bravo, Jordi Deu-Pons, Anna Bauer-Mehren, Martin Baron, Ferran Sanz, Laura I. Furlong, DisGeNET: a discovery platform for the dynamical exploration of human diseases and their genes *Database*, Volume 2015, 2015, bav028,https://doi.org/10.1093/database/bav028) integrates expert-curated databases with text-mined data, covers information on Mendelian and complex diseases, and includes data from animal disease models. It is a good choice for complex disease research, and it contains many important lupus studies based on the supporting evidenceto prioritize gene-and lupus associations.

### 2.4 RNA Extraction and PCR

RNA extraction: Fasting ethylenediaminetetraacetic acid (EDTA)-anticoagulated peripheral blood (2 mL) was collected from SLE patients and controls and was tested within 3 h. Ficoll separating solution was used to extract peripheral-blood mononuclear cells (PBMCs), lysis buffer was added to the extracted human PBMCs, the solution was placed at room temperature for 5 min, and 0.2 mL chloroform was added (2). Complementary DNA (cDNA) synthesis: The mixture was prepared in RNase-free microfuge tubes using a cDNA reverse transcription kit (Shanghai Yisheng Biotechnology Co., Ltd.), and the reverse transcription reaction system was prepared (20 μL) (3). PCR: According to the qPCR SYBR Green reagent kit, 2× real-time quantitative PCR amplification premixed solution, upstream and downstream primers, template DNA, and sterile ultrapure water were added. The reaction was performed in a fluorescence quantitative PCR machine (Thermo Scientific). The fluorescence quantitative PCR primer sequences are listed in **Supplementary Table 1**. According to the Ct value of the samples, the relative quantitative method was used to analyze the results of RT-PCR, and 2^-ΔCt^ was calculated (32).

## 3 Statistical analysis

SPSS 17.0 software was used to analyze the data. Measurement data with a normal distribution are expressed as the mean ± standard deviation (x ± S). The t test was used for intergroup comparison, and the paired t test was used for intragroup comparison. Measurement data with a nonnormal distribution are represented by the median (interquartile range), and the rank test was used for their intergroup and intragroup comparisons. Correlations between two variables were calculated using linear correlation analysis. Receiver operating characteristic (ROC) curve analysis was done to evaluate the sensitivity and specificity of CDC27 expression in PBMCs for SLE diagnosis. *P*<0.05 was considered statistically significant.

## 4 Results

### 4.1 Included Lupus Families

**Figure 3:**
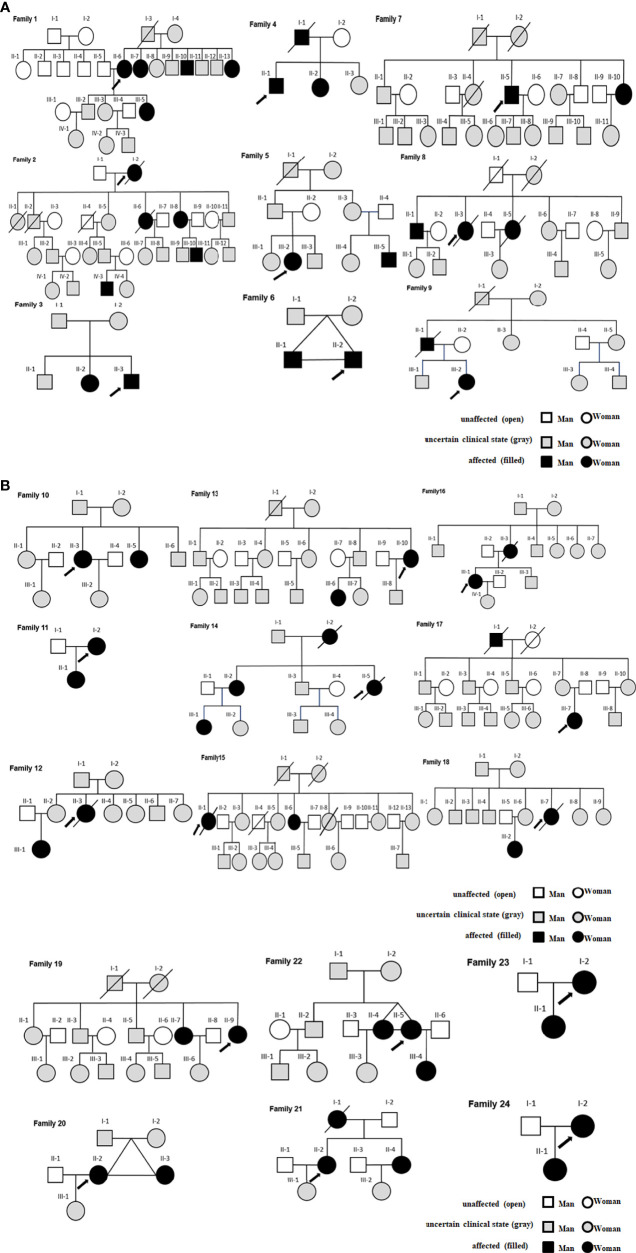
**(A)** Class 1-SLE families. **(B)** Class 2-SLE families.

There were 9 families in class 1, and 15 families in class 2 (**Figure 3**). CDC27 was involved in 10 families (see the family10-20 in **Figure 3** for details). Gray refers to people with uncertain lupus disease; open refers to healthy controls; filled refers to lupus patients in **Figure 3**.

### 4.2 Selected Candidate Genes

The two types of families, class 1 and class 2, were analyzed by three methods (common-specific analysis, pVAAST multiline linkage and correlation analysis, and Exomiser). The screening results are shown in **Supplementary Table 2**.

### 4.3 FARVAT Results

In addition to the above three analytical methods, FARVAT was used to analyze all the families. The results showed that only CDC27 was detected at the extremely significant level (*P*<0.05) in three burden analyses (BURDEN, CALPHA, and SKATO), as shown in **Table 1**.

**Table 1:** Results of family-based analysis using three types of burden (BURDEN, CALPHA, SKATO).

CHR	GENE	NSAMP	NVARIANT	MAC	NIMP	START	END	P-CALPHA	P-BURDEN	P-SKATO
17	CDC27	74	138	1969	8168	45198086	45266730	0.011191	0.007543	0.007507

NSAMP, Number of samples; “NVARIANT”, variance.

### 4.4 Annotation of Candidate Genes in the DisGenet Database

The candidate genes obtained from common-specific analysis, pVAAST multiline linkage and correlation analysis, FARVAT, and Exomiser pathogenic gene screening were annotated in the DisGenet database. Some genes in the database are reportedly related to lupus, as shown in **Supplementary Tables 3** **-** **6**.

### 4.5 Relationship Between the CDC27 Expression in PBMCs and Clinical Manifestations in SLE Patients

**Figure 4:**
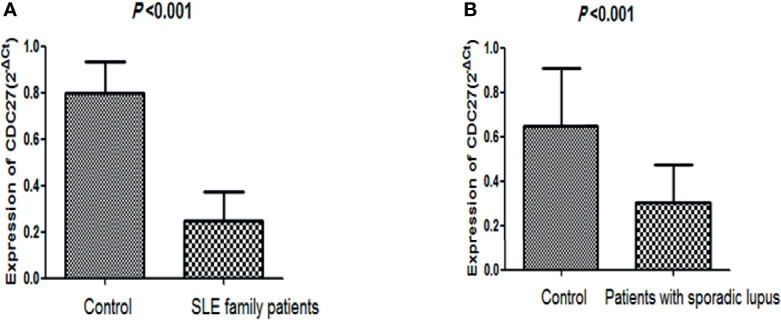
Comparison of CDC27 expression levels between healthy controls and lupus patients. **(A)** Comparison of healthy controls and family patients with lupus; **(B)** comparison of healthy controls and sporadic lupus patients.

There were no significant differences in sex, age, or body mass index between the two groups (sporadic lupus and healthy control group), nor between the familial lupus patients and the familial control group. QPCR was performed to detect for CDC27 in the PBMCs of the SLE familial patients, sporadic lupus patients, and healthy people. In the lupus family study of 15 patients with lupus and 14 healthy controls, CDC27 expression was lower in the patients than in the healthy controls, as depicted in **Figure 4A**. In the sporadic lupus analysis involving 92 patients and 48 healthy controls, CDC27 expression was also lower in the patients than in the healthy controls, as shown in **Figure 4B**.

**Figure 5:**
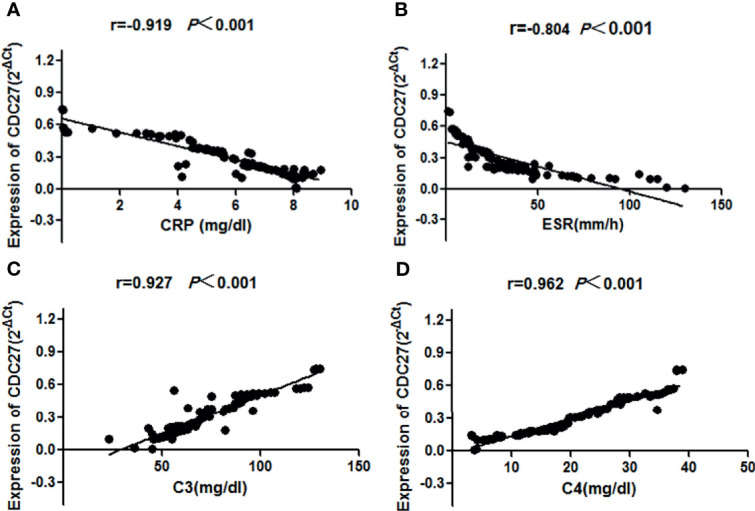
Correlations between the SLE disease activity index and CDC27 expression were calculated using linear correlation analysis **(A)** CRP, **(B)** ESR, **(C)** C3, **(D)** C4.

The clinical manifestations and laboratory tests of 107 SLE patients including 92 sporadic and 15 familial patients were collected in detail, and the SLE disease activity index (SLEDAI) was calculated (33). Clinical manifestations and laboratory indicators, such as erythrocyte sedimentation rate, C-reactive protein (CRP), complement C3, complement C4, antinuclear antibodies, double-stranded DNA, proteinuria, and hematuria, were examined to analyze the relationship between these indicators and the CDC27 expression in SLE patient PBMCs. The results showed that the CDC27 expression in PBMCs correlated negatively with CRP (r=-0.919, *P*<0.01), erythrocyte sedimentation rate (r=–0.804, *P*<0.001), C3 (r=0.927, *P*<0.001) and C4 (r=0.962, *P*<0.001), but not with the remaining indicators. Details are provided in **Figure 5**.

### 4.6 Relationship Between the CDC27 Expression in PBMCs of SLE Patients and Immunosuppressive Therapy

Among the 100 sporadic SLE patients enrolled, 18 exhibited decreased disease activity after immunosuppressive therapy (*P*<0.05), and samples before and after the activity changes were collected for CDC27 detection in PBMCs. CDC27 expression was upregulated after treatment, a difference that was statistically significant (*P* < 0.05), as shown in **Table 2**.

**Table 2:** The relationship between the expression of CDC27 in PBMC of SLE patients and immunosuppressive therapy.

Progect	Before immunotherapy	After immunotherapy	*P*
SLE Activity Score	10.944±2.127	4.778±1.833	*P*<0.001
CDC27 expression	0.3081±0.217	0.8966±0.981	0.023

### 4.7 Evaluation of the Diagnostic Value of CDC27 Expression in PBMCs for the SLE Patients

**Figure 6:**
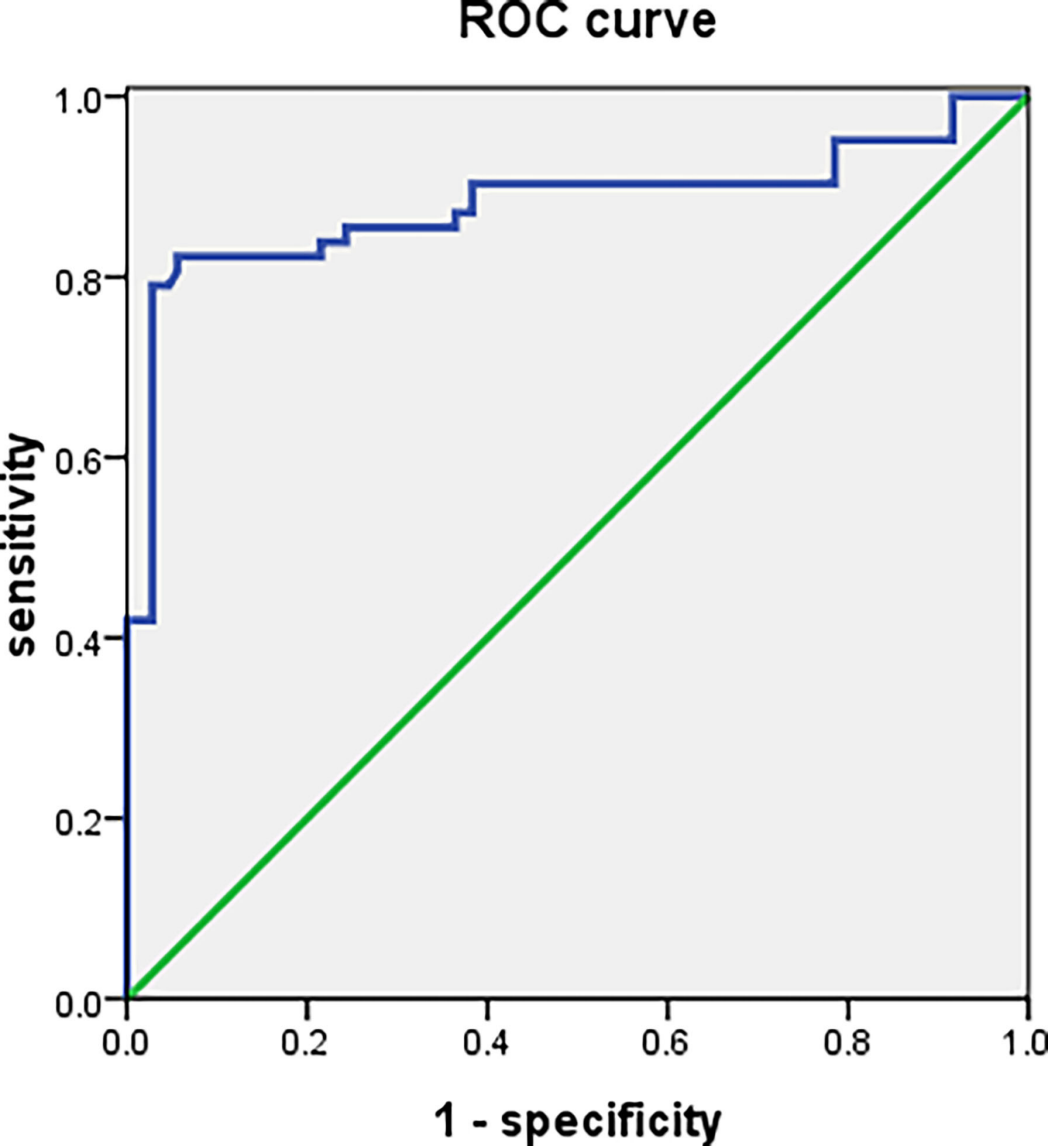
ROC curve analysis for sporadic lupus patients and healthy controls.

The CDC27 expression in PBMCs from107 SLE patients and 48 controls was analyzed by ROC analysis. The area under the ROC curve (AUC) was 0.880 (95% CI: 0. 814 <0. 946; P ~0. 001), the sensitivity was 82.30%, the specificity was 94.40% and the Youden index was 0.767. The positive predictive value is 90.09% and the negative predictive value is 89.47%.Details are shown in **Figure 6**.

## 5 Discussion

To date, a variety of genetic abnormalities have been found to be closely related to the occurrence and progression of SLE (34). However, there are few studies on causative genes obtained through pedigrees (35), especially on nonconsanguineous pedigrees through sequencing techniques.

In pedigree studies, WES is not limited to small pedigrees, multiple generations of inherited pedigrees, or core pedigrees with polygenic complex diseases, and it can create conditions to find causative genes (36). In this study, for the first time, many Chinese familial SLE patients (a total of 24 families) were sequenced using WES, and common-specific analysis, pVAAST multiple-line linkage and correlation analysis, FARVAT, and Exomiser were used to screen the causative genes of lupus families in order to screen for SLE causative genes through a pedigree study, especially SLE pedigrees of nonclose relatives. Common-specific analysis, pVAAST multiline linkage and correlation analysis, and Exomiser were used to screen genes and their corresponding loci. Common-specific analysis is a widely used strategy for patients with familial diseases. As the pathogenic loci of the corresponding disease may be enriched in the family, pVAAST was used. Exomiser considers the correlation between the phenotypes and genes found in humans and animal models and combines protein–protein interactions and other information, giving it an advantagefor locus the screening. When a certain gene is screened out by multiple methods, the reliability and scientific support for its causative role is high. FARVAT can be used for multiple pedigrees; it analyzes gene burden using the gene as a unit, and directly considers some genes with weak single-locus effect values. Thus, it is a supplement to the above three methods. In this study, the four analytical methods were combined to improve the reference value for screening causative genes of SLE through pedigrees.

Genetic factors play a significant role in the occurrence and progression of many diseases, such as coronary heart disease (37, 38), diabetes (39) and diabetes insipidus (40). However, it is very difficult to screen causative genes through pedigrees, and there is no unified and standardized method. Various methods have shortcomings, i.e., some can only perform enrichment (41), some can only find regions of causative genes, it is difficult to clearly identify specific genes in studies from small pedigrees (42) some pedigrees are difficult to screen due to incomplete data (43), and many related studies have been conducted on the consanguineous pedigrees. This study also provides a method for screening other diseases through pedigrees, especially consanguineous pedigrees (**Figure 1**).

We annotated the candidate genes in the DisGenet database. Much of the information we found is related to lupus, which indicates that the candidate genes in this study are important in the pathogenesis of lupus and have a certain degree of credibility. Among the four bioinformatic analyses, CDC27 was obtained using three methods. Therefore, we focused on this gene.

The major functional isomers of CDC27 are encoded by 19 exons, and CDC27 has two tetratricopeptide repeat (TPR) domains, five TPR motifs at the N-terminal domain and nine TPR motifs in the C-terminal domain (44). The protein encoded by CDC27 is a component of APC, which had a TPR sequence of its own that is necessary for interaction with certain proteins (45). CDC27 expression is abnormal in several tumors and autoimmune diseases (19), and their pathogenesis may be related to APC/C activation. Phosphorylation of CDC27 is the key for APC/C activation, which is achieved through the action of transforming growth factor β (TGF-β) (46). The TGF-β signaling pathway plays an important role in many biological processes, including cell growth, differentiation, apoptosis, and migration, and the occurrence and progression of autoimmune diseases (47). The activation of the TGF-β pathway is involved in the pathogenesis of SLE (48). Vanarsa et al. found that expression of TGF-β1 in the urine was increased in patients with active lupus (49).

To date, there had been no direct study on the relationship between CDC27 and lupus. In the present study, peripheral blood of familial SLE patients and healthy controls was submitted to qPCR to detect CDC27, and the results showed low CDC27 expression in lupus patients. We speculate that the downregulation of CDC27 in lupus may lead to immune disorders. Because CDC27 is a downstream molecule of the TGF-β pathway, it activates APC/C (ubiquitin ligase) under the action of TGF-β through phosphorylation, thereby activating the ubiquitin-proteasome system, which may affect the cell cycle process, hinder protein degradation, and cause immune abnormalities. The mechanism of abnormal CDC27 expression in lupus still needs to be further explored.

Diagnosis of SLE is mainly based on clinical symptoms, signs, and laboratory tests. However, the clinical manifestations of SLE are complex, and the severity of the disease varies; hence, clinicians often misdiagnose SLE as other diseases. The diagnostic sensitivity of commonly used autoantibodies (such as antinuclear antibodies, double-stranded DNA, and anti-Smith antibody) against SLE is not perfectat only 20-60% (50). Thus, there is no ideal diagnostic marker for SLE. This study used ROC curve analysis to assess the diagnostic value of CDC27 and found a sensitivity of 82.30%,and specificity of 94.40%,indicating that CDC27 has high value in the diagnosis of SLE (51). In addition, the expression level of CDC27 correlated with the activity of lupus and had certain value in predicting the condition.

Although we screened for abnormal expression of CDC27 in SLE patients through pedigrees, this gene’s role is not limited to familial SLE. To further investigate the general role of abnormal CDC27 expression in the pathogenesis of SLE, we detected CDC27 expression in patients with sporadic SLE and compared it with that in the healthy control. As expected, CDC27 expression in sporadic SLE patients was reduced, indicating that family-based causative gene exploration is not be limited to familial diseases, and that decreased expression of CDC27 has significance for the diagnosis of both familial and sporadic SLE. This study has reference value for research methods that study diseases through pedigrees to find patterns and apply them to the whole population.

## 6 Conclusion

For the first time, WES combined with a variety of analytical methods showed that the CDC27 gene plays an important role in the occurrence and progression of SLE and may be a causative gene and a marker of the disease. This study performed a preliminary verification, but further verification is still needed. We hope that the analytical method described herein provide new ideas for screening causative genes of other diseases through small pedigrees.

## Data Availability Statement

The data presented in the study are deposited in the https://bigd.big.ac.cn/gsa-human/browse repository, accession number HRA002049.

## Ethics Statement

The studies involving human participants were reviewed and approved by the Ethics Committee of the People’s Liberation Army General Hospital, and the ethics number is S2019-095-01. The patients/participants provided their written informed consent to participate in this study.

## Author Contributions

SS, XC, SC, and QL designed the study. SS and YZ wrote the original draft. SS, PL, XC, LC and QL provided the patient plasma samples and the corresponding clinical data. SS and MZ validated and interpreted the data. SS, KC and DL did the statistical analyses. MZ provided the code (https://github.com/zmj168/genetics). All authors contributed to the article and approved the submitted version.

## Funding

This study was funded by the National Natural Science Foundation of China (No. 81830019) and Beijing Natural Science Foundation(7202188).

## Conflict of Interest

Author LC was employed by Beijing Mygenostics co., LTD and author MZ was employed by Geneis (Beijing) Co., Ltd., Beijing, China.

The remaining authors declare that the research was conducted in the absence of any commercial or financial relationships that could be construed as a potential conflict of interest.

## Publisher’s Note

All claims expressed in this article are solely those of the authors and do not necessarily represent those of their affiliated organizations, or those of the publisher, the editors and the reviewers. Any product that may be evaluated in this article, or claim that may be made by its manufacturer, is not guaranteed or endorsed by the publisher.

## Abbreviations

dsDNA, Anti-double-stranded DNA; C3, Complement protein C3; C4, Complement protein C4; SLE, Systemic lupus erythematosus; SLEDAI, Systemic Lupus Erythematosus Disease Activity Index.
