# Supplementary material for: A Novel Gene CDC27 Causes SLE and Is Associated With the Disease Activity
Source: Front Immunol. 2022 Mar 28;13:876963. doi: 10.3389/fimmu.2022.876963 (PMC8996071; doi:10.3389/fimmu.2022.876963)
Supplement: Supplementary file 1 [file Table_1.docx]

Supplementary table 1.The fluorescence quantitative PCR primer sequences

| Gene | Primer sequence |
| --- | --- |
| CDC27 | CCGCTCAGGAAAGGCATATAAA  AGGTATTTGCATTGCGGTGTA |
| GAPDH | CTGGGCTACACTGAGCACC  AAGTGGTCGTTGAGGGCAATG |
