# Supplementary material for: A Novel Gene CDC27 Causes SLE and Is Associated With the Disease Activity
Source: Front Immunol. 2022 Mar 28;13:876963. doi: 10.3389/fimmu.2022.876963 (PMC8996071; doi:10.3389/fimmu.2022.876963)
Supplement: Supplementary file 2 [file Table_2.docx]

| Gene | Common-specific analysis | | Exomiser | | pVAAST | |
| --- | --- | --- | --- | --- | --- | --- |
|  | ClassI | ClassII | ClassI | ClassII | ClassI | ClassII |
| CDC27 |  | + |  | + |  |  |
| TTN | + |  | + |  |  |  |
| IGSF3 |  | + |  |  | + |  |
| PRKDC | + |  |  |  | + |  |
| ZFHX3 | + |  | + |  | + |  |
| MUC16 |  | + |  |  |  | + |
| DNAH8 | + |  |  |  | + |  |

Supplementary table 2 Genes screened by different analytical methods

+, represents the method and population of the screened genes;
