# Supplementary material for: A Novel Gene CDC27 Causes SLE and Is Associated With the Disease Activity
Source: Front Immunol. 2022 Mar 28;13:876963. doi: 10.3389/fimmu.2022.876963 (PMC8996071; doi:10.3389/fimmu.2022.876963)
Supplement: Supplementary file 3 [file Table_3.docx]

Supplementary table 3. Annotation of candidate genes in the DisGenet database（Screened genes for common-specific analysis）

| Ttype | DiseaseID | Disease_Term | CCount | Gene |
| --- | --- | --- | --- | --- |
| CClassI | C0024141 | Lupus Erythematosus, Systemic | 4 | ATN1;0.03\|APC;0.01\|C6;0.01\|PRKDC;0.01 |
|  | C0024138 | Lupus Erythematosus, Discoid | 2 | APC;0.01\|ATN1;0.01 |
|  | C0409974 | Lupus Erythematosus | 2 | APC;0.01\|ATN1;0.01 |
|  | C0024131 | Lupus Vulgaris | 2 | APC;0.01\|ATN1;0.01 |
|  | C0024143 | Lupus Nephritis | 1 | NOTCH3;0.01 |
|  | C0311370 | Lupus anticoagulant disorder | 1 | APC;0.01 |
| CClassII | C0024141 | Lupus Erythematosus, Systemic | 4 | LYST;0.1\|ATXN1;0.1\|HLA-DRB5;0.01\|RSPH6A;0.01 |
