# Supplementary material for: A Novel Gene CDC27 Causes SLE and Is Associated With the Disease Activity
Source: Front Immunol. 2022 Mar 28;13:876963. doi: 10.3389/fimmu.2022.876963 (PMC8996071; doi:10.3389/fimmu.2022.876963)
Supplement: Supplementary file 4 [file Table_4.docx]

Supplementary table 4. Annotation of candidate genes in the DisGenet database（Screened genes for pVAAST）

| Type | DiseaseID | Disease_Term | Count | Gene |
| --- | --- | --- | --- | --- |
| ClassI | C0024141 | Lupus Erythematosus, Systemic | 6 | C1R;0.3\|NOTCH4;0.11\|ANKS1A;0.1\|PRKDC;0.01\|TNS1;0.01\|LAMA1;0.01 |
|  | C0024138 | Lupus Erythematosus, Discoid | 2 | C1R;0.1\|TNS1;0.01 |
|  | C0024131 | Lupus Vulgaris | 1 | TNS1;0.01 |
|  | C0024143 | Lupus Nephritis | 1 | TNS1;0.01 |
|  | C0409974 | Lupus Erythematosus | 1 | TNS1;0.01 |
| Class II | C0024141 | Lupus Erythematosus, Systemic | 3 | ATXN1;0.1\|CCR5;0.03\|VWF;0.02 |
|  | C0409974 | Lupus Erythematosus | 2 | CCR5;0.01\|VWF;0.01 |
|  | C0024138 | Lupus Erythematosus, Discoid | 2 | CCR5;0.01\|VWF;0.01 |
|  | C0024131 | Lupus Vulgaris | 2 | CCR5;0.01\|VWF;0.01 |
|  | C0024143 | Lupus Nephritis | 1 | CCR5;0.06 |
|  | C0311370 | Lupus anticoagulant disorder | 1 | VWF;0.01 |
