# Supplementary material for: A Novel Gene CDC27 Causes SLE and Is Associated With the Disease Activity
Source: Front Immunol. 2022 Mar 28;13:876963. doi: 10.3389/fimmu.2022.876963 (PMC8996071; doi:10.3389/fimmu.2022.876963)
Supplement: Supplementary file 5 [file Table_5.docx]

Supplementary table 5．Annotation of candidate genes in the DisGenet database（Genes screened by Exomiser）

| Type | DiseaseID | Disease_Term | Count | | Gene |  |
| --- | --- | --- | --- | --- | --- | --- |
| ClassI | C0024141 | Lupus Erythematosus, Systemic | 9 | | SIGLEC6;0.3\|RFX1;0.03\|APC;0.01\|ATM;0.01\|GGT1;0.01\|NUP98;0.01\|PRKDC;0.01\|GGTLC3;0.01\|  GGT2;0.01 |  |
|  | C0409974 | Lupus Erythematosus | 2 | | RFX1;0.03\|APC;0.01 |  |
|  | C0024138 | Lupus Erythematosus, Discoid | 2 | | RFX1;0.03\|APC;0.01 |  |
|  | C0024131 | Lupus Vulgaris | 2 | | RFX1;0.03\|APC;0.01 |  |
|  | C0311370 | Lupus anticoagulant disorder | 1 | | APC;0.01 |  |
|  | C0024143 | Lupus Nephritis | 1 | | CIC;0.01 |  |
|  |  | Lupus Erythematosus, Discoid | 1 | | ATN1;0.01 |  |
|  | C0024131 | Lupus Vulgaris | 1 | | ATN1;0.01 |  |
|  | C0024141 | Lupus Erythematosus, Systemic | 1 | | ATN1;0.03 |  |
|  | C0409974 | Lupus Erythematosus | 1 | ClassII | | C0024138 |
| ClassII | C0024141 | Lupus Erythematosus, Systemic | 14 | HLA-DRB1;0.4\|ITGAX;0.13\|LPP;0.1\|ACE;0.09\|CD44;0.06\|KIR3DL1;0.03\|  CREBBP;0.02\|FN1;0.01\|GGT1;0.01\|KIR2DL1;0.01\|PKLR;0.01\|RREB1;0.01\|OSMR;0.01\|GGTLC3;0.01 | |  |
|  | C0024131 | Lupus Vulgaris | 6 | HLA-DRB1;0.03\|ACE;0.02\|CD44;0.01\|CREBBP;0.01\|KIR3DL1;0.01\|PKLR;0.01 | |  |
|  | C0409974 | Lupus Erythematosus | 6 | HLA-DRB1;0.03\|ACE;0.02\|CD44;0.01\|CREBBP;0.01\|KIR3DL1;0.01\|PKLR;0.01 | |  |
|  | C0024138 | Lupus Erythematosus, Discoid | 6 | HLA-DRB1;0.03\|ACE;0.02\|CD44;0.01\|CREBBP;0.01\|KIR3DL1;0.01\|PKLR;0.01 | |  |
|  | C0024143 | Lupus Nephritis | 5 | | ACE;0.04\|FN1;0.02\|HLA-DRB1;0.02\|CD44;0.01\|MYH9;0.01 |  |
|  | C0024140 | Lupus Erythematosus, Subacute Cutaneous | 1 | | HLA-DRB1;0.01 |  |
|  | C0311370 | Lupus anticoagulant disorder | 1 | | HLA-DRB1;0.01 |  |

4. Annotation of candidate genes in the DisGenet database（FARVAT screened genes）

| Type | DiseaseID | Disease_Term | Count | Gene |
| --- | --- | --- | --- | --- |
| ClassII | C0024141 | Lupus Erythematosus, Systemic | 4 | NADSYN1;0.1\|BCR;0.04\|EZH2;0.02\|BSG;0.01 |
|  | C0409974 | Lupus Erythematosus | 2 | BCR;0.01\|EZH2;0.01 |
|  | C0024138 | Lupus Erythematosus, Discoid | 2 | BCR;0.01\|EZH2;0.01 |
|  | C0024141 | Lupus Erythematosus, Systemic | 4 | ASS1;0.01\|BSG;0.01\|CGB3;0.01\|CFHR3;0.01 |
