# Supplementary material for: A Novel Gene CDC27 Causes SLE and Is Associated With the Disease Activity
Source: Front Immunol. 2022 Mar 28;13:876963. doi: 10.3389/fimmu.2022.876963 (PMC8996071; doi:10.3389/fimmu.2022.876963)
Supplement: Supplementary file 6 [file Table_6.docx]

Supplementary table 6. Annotation of candidate genes in the DisGenet database（FARVAT screened genes）

| Type | DiseaseID | Disease_Term | Count | Gene |
| --- | --- | --- | --- | --- |
| ClassII | C0024141 | Lupus Erythematosus, Systemic | 4 | NADSYN1;0.1\|BCR;0.04\|EZH2;0.02\|BSG;0.01 |
|  | C0409974 | Lupus Erythematosus | 2 | BCR;0.01\|EZH2;0.01 |
|  | C0024138 | Lupus Erythematosus, Discoid | 2 | BCR;0.01\|EZH2;0.01 |
|  | C0024141 | Lupus Erythematosus, Systemic | 4 | ASS1;0.01\|BSG;0.01\|CGB3;0.01\|CFHR3;0.01 |
